# Study on the Pauropoda (Myriapoda) from Tibet, China – Part II: New species and new record of the genus *Samarangopus*

**DOI:** 10.3897/zookeys.927.50100

**Published:** 2020-04-16

**Authors:** Yun Bu

**Affiliations:** 1 Natural History Research Center, Shanghai Natural History Museum, Shanghai Science & Technology Museum, Shanghai 200041, China

**Keywords:** appendages, Eurypauropodidae, Motuo County, pauropod, taxonomy

## Abstract

The pauropod family Eurypauropodidae Ryder, 1879 is recorded from Tibet, China for the first time. In this study, a new species *Samarangopus zhongi***sp. nov.** is described and illustrated from Motuo County, southeastern Tibet of China. It is distinguished from other species in this genus by having one pair of spiniform appendages on the sternum of the last trunk segment, 28–34 marginal protuberances on tergite I, the distal quarter of bothriotricha *T*_3_ golf-club-shaped, and the leaf-shaped seta *st* on tergum of pygidium. In addition, *Samarangopus canalis* Scheller, 2009 is newly recorded from China.

## Introduction

Four species of pauropods were recognized in Tibet: *Sphaeropauropus* sp. belonging to the family Sphaeropauropodidae Silvestri, 1930 (Zhang and Chen 1988), *Decapauropus biconjugarus* Qian & Bu, 2018, *D. tibeticus* Qian & Bu, 2018, and *Hemipauropus quadrangulus* Qian & Bu, 2018 (Qian et al. 2018) belonging to the family Pauropodidae Lubbock, 1867. However, the investigation of pauropod diversity in Tibet is still insufficient.

The family Eurypauropodidae Ryder, 1879 is currently comprised of more than 60 species (Scheller 2011). It is diagnosed by the following characters: 1) body flattened dorsoventrally; 2) entire tergites strongly sclerotized; 3) incapability to coil the body; 4) coarse and ornamented surface of tergites with modified setae and marginal protuberances. Only two species of Eurypauropodidae were so far reported from China: *Eurypauropus* sp. from Zhejiang Province (Zhang and Chen 1988) and *Samarangopus dilatare* Qian, 2014 from Jiangxi Province (Qian et al. 2014).

The purposes of this study are 1) to record the occurrence of family Eurypauropodidae Ryder, 1879 in Tibet; 2) to describe a new species of the genus *Samarangopus* Verhoeff, 1934; 3) to record the presence of *Samarangopus canalis* Scheller, 2009 in southeastern Tibet for the first time.

## Materials and methods

All pauropods were collected using a Tullgren’s funnel. The specimens were sorted under a stereomicroscope and preserved in 80% alcohol. They were mounted on slides using Hoyer’s solution and dried in an oven at 50 °C. Observations were performed under a phase contrast microscope (Leica DM 2500). Photos were taken using a digital camera (Leica DMC 4500). Line drawings were made using a drawing tube. All specimens were deposited in the collection maintained by the Shanghai Natural History Museum (**SNHM**).

Abbreviations used in the descriptions follow Qian et al. (2018). Absolute lengths of all other body parts are given in mm and μm. Otherwise, the text refers relative lengths. For the description of the new species, measurements and indices of paratypes are given in brackets.

## Results

### Taxonomy

**Family Eurypauropodidae Ryder, 1879**

#### Genus Samarangopus

Taxon classificationAnimaliaTetramerocerataEurypauropodidae

Verhoeff, 1934

B5851913-1301-5131-8AE7-BA6792BA917D

##### Type species.

*Samarangopus jacobsoni* (Silvestri, 1930).

##### Diagnosis.

Fourth antennal segment with 3 well developed setae; globulus of sternal antennal branch g short-stalked; all legs 5-segmented; empodia with 1 anterior accessory claw (Scheller 2011).

##### Distribution.

Palaearctic, Ethiopian, Oriental, and Australian regions.

#### Samarangopus zhongi sp. nov.

Taxon classificationAnimaliaTetramerocerataEurypauropodidae

D105419A-EC46-5461-B757-BBB0A01AFB2C

http://zoobank.org/8A4279A5-7389-45DC-A026-D3E523CA0F33

Figures 1 , 2 , 3

##### Material examined.

***Holotype***, male adult with 9 pairs of legs (slide no. XZ-PA2015004) (SNHM), China, Tibet, Motuo county, Dexing town, extracted from soil samples in a broad-leaf forest, alt. 1100 m, 29°40'N, 95°26'E, 3-XI-2015, coll. Y. Bu. ***Paratypes***, 5 male adults with 9 pairs of legs (slides no. XZ-PA2015001, XZ-PA2015006, XZ-PA2015052, XZ-PA2015056, XZ-PA2015057) (SNHM), 3 female adults, with 9 pairs of legs (slides no. XZ-PA2015005, XZ-PA2015024, XZ-PA2015054) (SNHM), same data as holotype. Other material, 1 juvenile, with 6 pairs of legs (slides no. XZ-PA2015051) (SNHM), same data as holotype.

##### Diagnosis.

*Samarangopus zhongi* sp. nov. is characterized by one pair of spiniform appendage on sternum of last trunk segment, 28–34 marginal protuberances on tergite I, the distal quarter of bothriotricha *T*_3_ golf-club-shaped, and the leaf-shaped seta *st* on tergum of pygidium.

##### Description.

**Figure 1.**
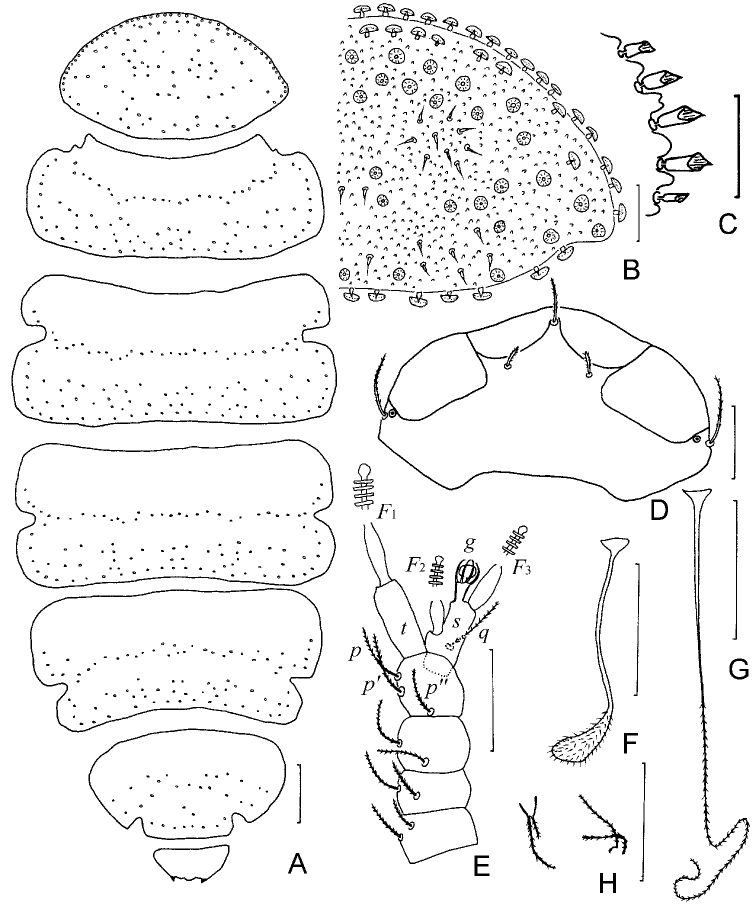
*Samarangopus zhongi* sp. nov. (holotype) **A** body, tergal view **B** tergite I, right side **C** protuberances on posterolateral corner of tergite I, right side **D** head, anteriodorsal view **E** left antenna, tergal view **F***T*_3_**G***T*_5_**H** setae on coxa (left) and trochanter (right) of leg 9. Scale bars: 20 μm.

Adult body length (0.62–) 0.69 (–0.75) mm (*n* = 9); body yellow to brown (Figs 1A, 3A).

***Head*** (Figs 1D, 3E) setae strongly reduced, dorsally with first row setae *a*_1_ and 1 pair of lateral setae, other setae absent. Temporal organs rectangular in tergal view, length 0.9 of shortest interdistance, glabrous. Tiny pistils present laterally.

***Antennae*** (Figs 1E, 3C). Chaetotaxy of segments 1–4: 2/2/2(g’)/3. Setae thin, cylindrical, striate, length of seate on segment 4: *p* =14 (–15) μm, *p*’ =14 (–17) μm, *p*’’ =12 (–15) μm; *u* and *r* absent. Tergal branch *t* cylindrical, (2.1–) 2.6 times as wide as greatest diameter and 1.0 (–1.1) times as long as sternal branch. Sternal branch *s* with distinct anterior indentation at level of *F2*, 1.9 (–2.3) times as long as greatest diameter, anterodistal corner distinctly truncate. Seta *q* similar to setae of segment 4, 15 (–17) μm, (0.9 of–) 1.1 times as long as the length of *s.* Globulus *g* with conical stalk, length of *g* (8–10 μm) 1.7 (–1.8) times as long as its greatest diameter; the latter 0.3 (–0.4) of greatest diameter of *t*; 10 bracts, capsule spherical, diameter = 4–5 μm; stalk length 4–5 μm. Relative lengths of flagella (base segments included): *F*_1_ = 100, *F*_2_ = 48 (–55), *F*_3_ = (78–) 84 (–89). Lengths of base segments: *bs*_1_ = (10–) 12, *bs*_2_ = 5 (–7), *bs*_3_ = 10 (–11) μm. *F*_1_ (4.1–) 4.4 times as long as *t*, *F*_2_ and *F*_3_ (1.9–) 2.3 and (3.2–) 3.7 times as long as sternal branch *s*, respectively. Calyces of *F*_1_ largest, conical, those of *F*_2_ and *F*_3_ smaller, subhemispherical.

**Figure 2.**
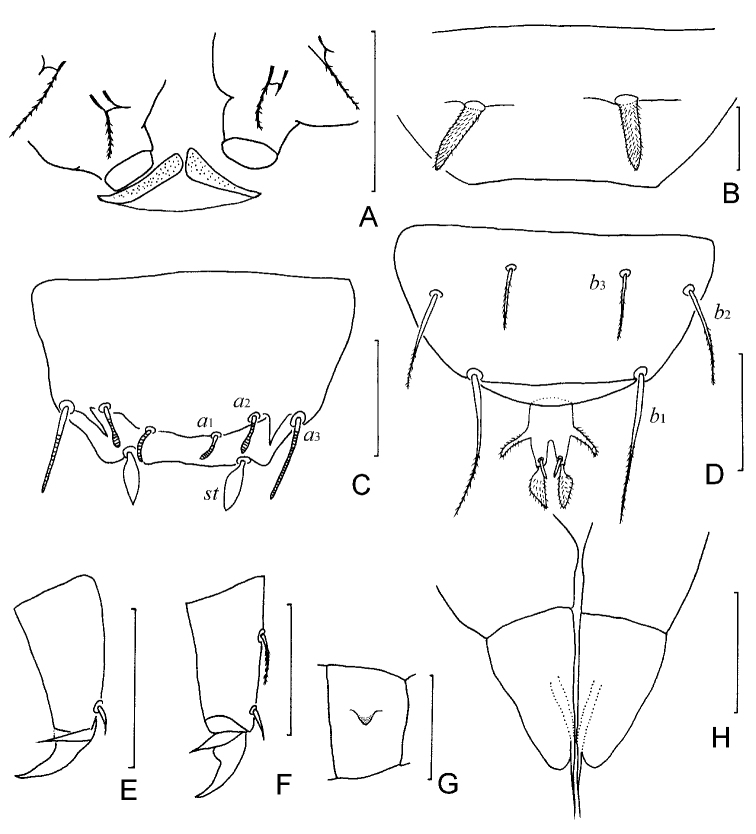
*Samarangopus zhongi* sp. nov. (holotype) **A** collum segment, sternal view **B** sternum of the last trunk segment, show spiniform appendages **C** tergum of pygidum **D** sternum of pygidum and anal plate **E** tarsus of leg 1 **F** tarsus of leg 9 **G** femur of leg 1 with appendage **H** male genital papillae. Scale bars: 20 μm.

**Figure 3.**
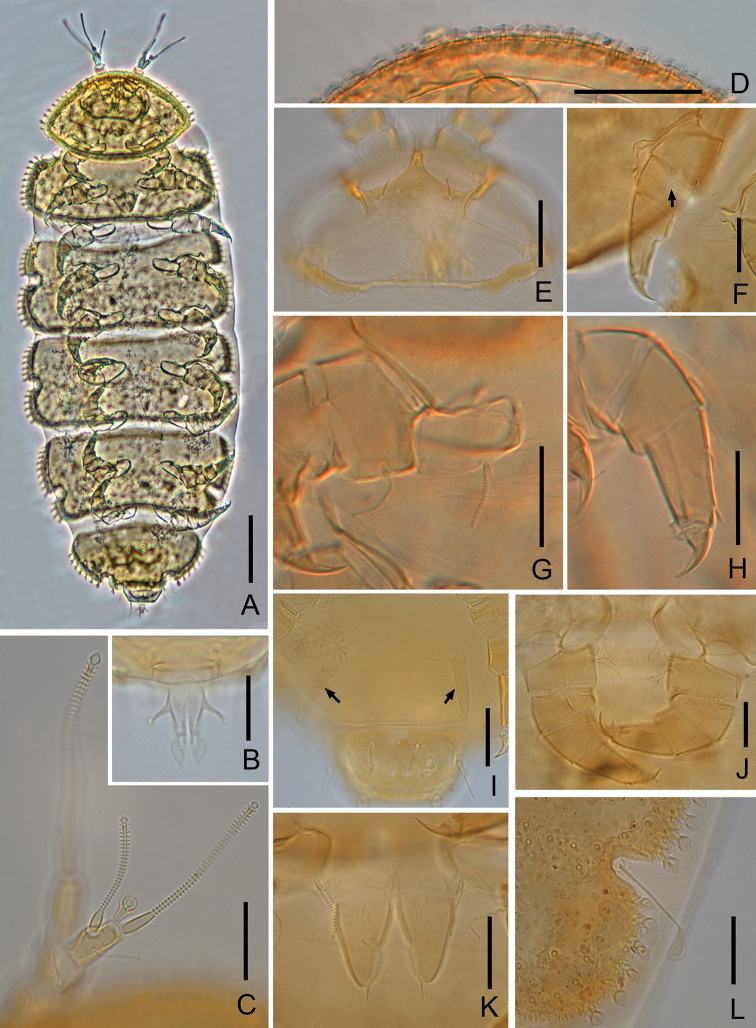
*Samarangopus zhongi* sp. nov. (holotype) **A** habitus, sternal view **B** anal plate **C** right antenna, sternal view **D** anterior margin of tergite I **E** head, tergal view **F** leg 1, arrow shows appendage on femur **G** setae on coxa and trochanter of leg 1 **H** tarsus of leg 9 **I** last trunk segment, arrows show one pair of appendages, sternal view **J** setae on coxa and trochanter of leg 9 **K** male genital papillae and coxa of leg 2 **L** right side margin of tergite IV and *T*_3_. Scale bars: 100 μm (**A**); 20 μm (**B–L**).

***Trunk*.** Setae of collum segment similar, furcate, branches tapering, pointed; main branch striate; secondary branch rudimentary, glabrous; both setae length 10 (–11) μm (Fig. 2A). Appendages barrel-shaped; caps flat (Fig. 2A). Sternite process broad and low, with anterior V-shaped incision. Tergites densely covered with protuberances (Figs 1A–C, 3D, L). Three main types of protuberances observed: large and stalked protuberances present on anterior margin of tergite I and lateral margins of I–VI; smaller fungiform protuberances with transparent hat and subcylindrical foot; small cylindrical cuticular structures with distal candle flame-like vesicle surrounded by circular collar. Cuticles between these structures coarse. Number of marginal protuberances: I, (28–) 34; II, 1 small- *T*_1_-1 small-(9–10); III, 1 small-7- *T*_2_-l small-7; IV, 1 small-(7–8)- *T*_3_-l small-5; V, 1 small-(7–9)- *T*_4_-1small-3; VI, (6–8)- *T*_5_-l. Length/width ratio of tergites: I=0.56 (–0.6), II=0.35 (–0.37), III and IV = 0.38 (–0.43), V = 0.45 (–0.48), V = (0.54–) 0.57 (Fig. 1A). Sternum of last trunk segment with one pair of blunt, spiniform, pubescent posterior appendages (Figs 2B, 3I), 23 (–26) μm in length.

***Bothriotricha*.** All with thin axes and short almost erect pubescence, *T*_1_, *T*_2_, *T*_4_, and *T*_5_ with distal part curled (Fig. 1G), *T*_3_ shorter than others, with thicker axis and distal quarter flat, golf-club-shaped, densely pubescent (Figs 1F, 3L). Relative lengths of bothriotricha: *T*_1_ = 100, *T*_2_ = (91–) 94, *T*_3_ = (45–) 53, *T*_4_ = (100–) 106, *T*_5_ = 112 (–115).

***Legs*.** All legs 5-segmented. Setae on coxa and trochanter of leg 9 similar to each other, thin, furcate, striate, with glabrous base, length of secondary branch 0.7 (–0.8) of primary one (Figs 1H, 3J). On more anterior legs these setae similar to those of collum segment (Fig. 3G); Tarsi tapering, those of leg 9 (1.9–) 2.5 times as long as greatest diameter; proximal seta striate 11 μm, (0.4–) 0.5 of the length of tarsus (22–28 μm) and (1.7–) 2.0 times as long as distal glabrous seta (5–6 μm) (Figs 2F, 3H). Cuticle of tarsus glabrous (Fig. 3F–H). Tarsus of leg 1 with only glabrous distal seta (Fig. 2E). All legs with large main claw and small setose anterior secondary claw, the former on those of leg 9 0.5 of tarsi. On anterior side of femur of leg 1 with 1 blunt granulated appendage (Figs 2G, 3F).

***Genital papillae*** (Figs 2H, 3K). Base segments cylindrical. Length of papillae = 25 (–28) μm, greatest diameter =15 (–16) μm, length of seta= 9 (–11) μm. Proximal part of genital papillae subcylindrical, distal part conical, seta 0.4 (–0.5) of length of papilla. Cuticle glabrous. Coxal seta of leg 2 same as on leg 1 (Fig. 3G, K).

***Pygidium*. *Tergum*** (Fig. 2C). Posterior margin with 2 lateral triangular appendages between *a*_2_ and *a*_3_. Setae annulated, *a*_1_ and *a*_2_ somewhat clavate, the former curved inwards and the latter almost straight; *a*_3_ straight, cylindrical, somewhat tapering, diverging; *st* leaf-shaped, glabrous. Lengths of setae: *a*_1_= *a*_2_ =5 μm, *a*_3_ = 10 (–12) μm, *st* = 8 (–9) μm. Distances *a*_1_–*a*_1_=6 (–8) μm, *a*_1_–*a*_2_=5 (–6) μm, *a*_2_–*a*_3_=(4–) 5 μm, *st*–*st*=(8–) 10 μm.

***Sternum*** (Fig. 2D). Posterior margin between *b*_1_ straight. Setae thin, tapering, distally striate, pointed. Lengths of setae: *b*_1_= (25–) 28 (–30) μm, *b*_2_=17 (–20) μm, *b*_3_= (11–) 13 μm. Distance *b*_1_–*b*_1_= (26–) 27 μm, *b*_2_–*b*_2_= 45 (–47) μm, *b*_1_–*b*_2_=15 (–16) μm, *b*_3_–*b*_3_= (18–) 20 μm. *b*_1_ 1.0 (–1.3) times as long as interdistance, *b*_2_ (0.9 of–) 1.1 times as long as distance *b*_1_ –*b*_2_, *b*_3_ (0.6–) 0.7 of interdistance.

***Anal plate*** (Figs 2D, 3B) (2.1–) 2.2 times as long as broad, tapering posteriorly; lateral margins with a pair of thin, diverging, cylindrical, distal part faintly inflated, pubescent branches which are (0.3–) 0.4 of the length of plate; posterior 2/5 of plate divided into 2 tapering branches by a narrow V-shaped incision, each branch with 2 appendages: a submedian short, straight, tapering, glabrous one and a stalked bladder of triangular shape in sternal view. Bladder-shaped appendages (0.6–) 0.7 of length of plate. Plate glabrous, bladder-shaped appendages with short erect pubescence.

##### Etymology.

The new species is dedicated in honor of the late Professor Zhong Yang (1964–2017) who was an eminent botanist from Fudan University and Tibet University, for his great contribution to the knowledge of flora and biodiversity of Tibet. This study is also to express my great gratitude to his help.

##### Distribution.

Known only from the type locality.

##### Remarks.

*Samarangopus zhongi* sp. nov. can be easily distinguished from all other congeners by the presence of the one pair of spiniform appendages on the sternal side of last trunk segment. It is most similar to *S. campanulatus* Scheller, 2004 from Vietnam in the shape of anal plate, the chaetotaxy of pygidium and the protuberances on the body. It can be distinguished from *S. campanulatus* by: the spiniform appendages on the sternum of last trunk segment (present in *S. zhongi* sp. nov. vs absent in *S. campanulatus*), shape of bothriotricha *T*_3_ (distal quarter golf-club-shaped, densely pubescent in *S. zhongi* sp. nov. vs distal 2/5 part clavate, end-swelling in *S. campanulatus*), shape of seta *st* on tergum of pygidium (leaf-shaped in *S. zhongi* sp. nov. vs lanceolate in *S. campanulatus*), the shape of proximal seta on tarsus 9 (striated in *S. zhongi* sp. nov. vs glabrous in *S. campanulatus*), and the shape of appendage on the femur of leg 1 (broad triangular in *S. zhongi* sp. nov. vs blunt cylindrical in *S. campanulatus*) . The shape of posterior appendage on anal plate of the new species is also similar to *S. tuberosus* Scheller, 2007 from Singapore and *S. cylindratus* Scheller, 2009 from Indonesia. The new species differs from *S. tuberosus* in the shape of setae *b*_2_ on pygidium (slender and pointed in *S. zhongi* sp. nov. vs large and lanceolate in *S. tuberosus*). It differs from *S. cylindratus* in the shape of appendages of the collum segment (barrel-shaped in *S. zhongi* sp. nov. vs cylindrical and large in *S. cylindratus*).

#### Samarangopus canalis

Taxon classificationAnimaliaTetramerocerataEurypauropodidae

Scheller, 2009, new record to China

1D81096B-03FB-5F51-A5CD-058C53766994

Figure 4

##### Material examined.

1 male adult with 9 pairs of legs (slide no. XZ-PA2015053) (SNHM), 1 female adult with 9 pairs of legs (slide no. XZ-PA2015055) (SNHM), China, Tibet, Motuo county, Dexing town, extracted from soil samples of broad-leaf forest, Alt. 1100 m, 29°40'N, 95°26'E, 3-XI-2015, coll. Y. Bu.

##### Diagnosis.

*Samarangopus canalis* Scheller, 2009 is characterized by the peculiar shape of distal part of male genital papillae which forming an anteriorly open furrow and the ovoid posterior appendages of the anal plate.

##### Description of new materials.

**Figure 4.**
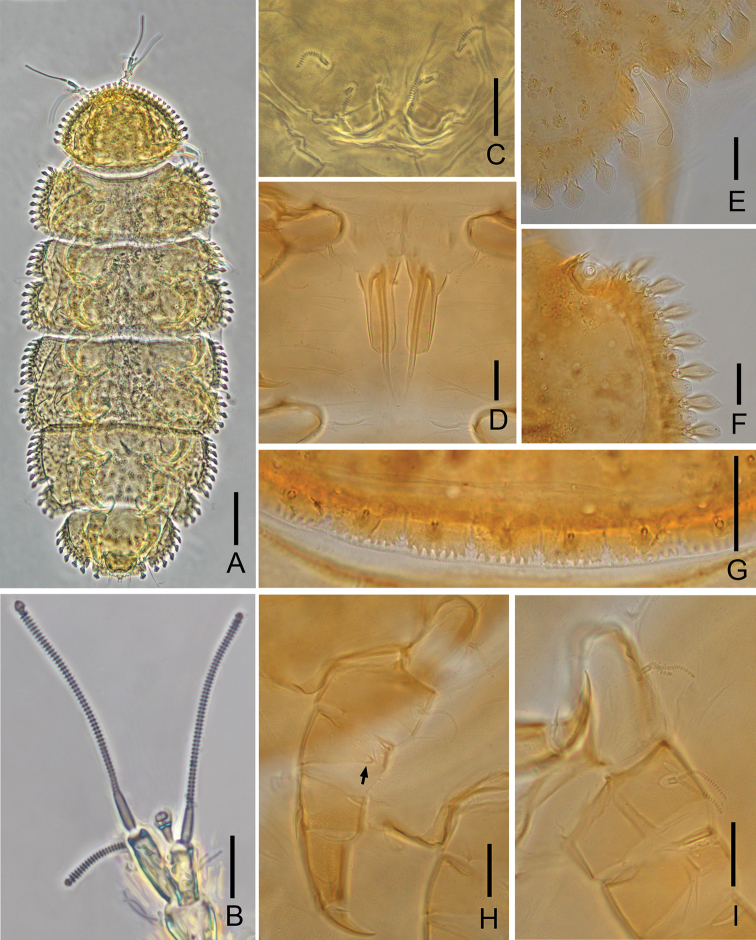
*Samarangopus canalis* Scheller, 2009 (Chinese specimens) **A** habitus, tergal view **B** left antenna, tergal view **C** collum segment, sternal view **D** male genital papillae **E** right margin of tergite IV, show *T*_3_**F** right anterolateral corner of tergite II, tergal view **G** posteriomedian margin of Tergite I **H** leg 1 **I** setae on coxa and trochanter of leg 9. Scale bars: 100 μm (**A**); 20 μm (**B–I**).

Length 0.90 mm (*n* = 2), yellow to brown in color (Fig. 4A). Head covered by tergite I and chaetotaxy not studied in detail.

***Antennae*** (Fig. 4B). Chaetotaxy of segments 1–4: 2/2/2/3. Setae thin, cylindrical, striate, length of seate on segment 4: *p* = 16–18 μm, *p*’ = 15–17 μm, *p*’’ = 10–12 μm; *p*’’’ rudimentary; *u* and *r* absent. Tergal branch *t* fusiform, 2.9–3.2 times as wide as greatest diameter and 1.2–1.3 times as long as sternal branch. Sternal branch *s* with distinct anterior indentation at level of *F*_2_, 1.8–2.0 times as long as greatest diameter, anterodistal corner distinctly truncate. Seta *q* similar to setae of segment IV, 15–16 μm, 0.8 of the length of *s.* Globulus *g* with conical stalk, length of *g* (10–12 μm) 1.7–2.0 times as long as greatest diameter; the latter 0.2–0.3 of greatest diameter of *t*; 9–10 bracts, capsule spherical, diameter = 3 μm; stalk length 5 μm. Relative lengths of flagella (base segments included): *F*_1_ = 100, *F*_2_ = 35–40, *F*_3_ = 82–84. Lengths of base segments: *bs*_1_ = 15–18 μm, *bs*_2_ = 7–8 μm, *bs*_3_ = 13–14 μm. *F*_1_ 3.3–3.7 times as long as *t*, *F*_2_ and *F*_3_ 1.6–1.9 and 3.6–3.9 times as long as sternal branch *s.* Calyces of *F*_1_ largest, conical, those of *F*_2_ and *F*_3_ smaller, subhemispherical.

***Trunk*.** Setae of collum segment similar, furcate, branches tapering, pointed; main branch cylindrical, annulated, blunt, secondary branch 0.3 of the length of primary branch, glabrous (Fig. 4C); submedian seta 0.8–0.9 of the length of sublateral seta. Sternite process broad and low, with anterior incision and rounded pubescent lobes. Appendages subcylindrical, caps flat. Process and appendages glabrous. Tergites densely covered with protuberances. Anterior and lateral margins of tergites with a single row of large protuberances (Fig. 4A, E, F). Posteriomedian margin of tergites with comb-shaped ornaments (Fig. 4G). Number of marginal protuberances: I, 26–29; II, 1 small- *T*_1_-1 small-9; III, (5–6)- *T*_2_-l small-6; IV, (6–7)- *T*_3_-l small-5; V, 8- *T*_4_-l small-3; VI, 6- *T*_5_-l. Length/width ratio of tergites: I = 0.67–0.72, II = 0.34–0.36, III and IV = 0.42–0.45, V = 0.44–0.46, VI = 0.56–0.59.

***Bothriotricha*.** All with thin axes, glabrous proximal parts, distally with minute pubescence, *T*_1_, *T*_2_, *T*_4_ and *T*_5_ curled distally, *T*_3_ shorter than others, with thicker axis and terminated by an ovoid swelling (Fig. 4E). Relative lengths of bothriotricha: *T*_1_ = 100, *T*_2_ = 94, *T*_3_ = 56, *T*_4_ = 94, *T*_5_ = 100.

***Genital papillae*** (Fig. 4D). Base segments in the shape of a truncated cone, relatively long, length of papillae 65 μm, greatest diameter 20 μm, seta 55 μm. Proximal part of papillae strongly tapering outward, distal 3/4 forming an anteriorly open furrow. Papilla 3.3 times as long as greatest diameter, seta 0.8 of length of papilla. Cuticle glabrous. Coxal seta of leg 2 in male with long base, furcate, primary branch cylindrical, annulated, secondary branch short, tapering, pointed, glabrous.

***Legs*.** All legs 5-segmented. Setae on coxa and trochanter of leg 9 similar to each other, thin, furcate, densely annulated, length of secondary branch 0.7–0.8 of primary one (Fig. 4I). On more anterior legs these setae with rudimentary secondary branches (Fig. 4H). Tarsi of leg 9 short and thick, tapering, 2.2 times as long as greatest diameter; tergal setae pointed, glabrous. Proximal seta length 9–10 μm, 0.3 of the length of tarsus (33 μm) and 1.2–1.3 times as long as distal seta (7–8 μm). Cuticle of tarsus with minute granules. No proximal seta on tarsus of leg 1. All legs with large main claw and small setose anterior secondary claw, the former on those of leg 9 0.5 of tarsi. Anterior side of femur of leg 1 with one blunt appendage with short pubescence, length = 4–5 μm (Fig. 4H).

***Pygidium*. *Tergum*.** Posterior margin with two narrow, digitiform posterior directed processes protruding from between setae *a*_2_ and *a*_3_. Setae glabrous, *a*_1_ straight, *a*_2_ clavate, short, *a*_3_ slender and long; *st* long and leaf-shaped, 10–11 μm. Two semicircle plates close to *st* with dense pubescence. Lengths of setae: *a*_1_= 5 μm, *a*_2_ =6–8 μm, *a*_3_ = 15 μm. Distance *a*_1_–*a*_1_=7–9 μm, *a*_1_–*a*_2_=5–7 μm, *a*_2_–*a*_3_=4–5 μm, *st*–*st*=10 μm.

***Sternum*.** Posterior margin between *b*_1_ almost straight. Setae thin, tapering, pointed, distal part of *b*_1_ annulated, *b*_2_ and *b*_3_ striated. Lengths of setae: *b*_1_= 32 μm, *b*_2_=23–25 μm, *b*_3_= 11–13 μm. Distances *b*_1_–*b*_1_= 28–30 μm, *b*_2_–*b*_2_= 50–53 μm, *b*_1_–*b*_2_= 21–23 μm, *b*_3_–*b*_3_= 23–25 μm. *b*_1_ 1.1 times as long as interdistance, *b*_2_ 1.0–1.2 of distance *b*_1_ –*b*_2_, *b*_3_ 0.55 of interdistance.

***Anal plate*.** 1.2 times as long as broad; lateral margins straight anteriorly, concave posteriorly; distal part of plate cleft by narrow U-shaped incision, depth 0.3–0.4 of the length of plate, incision forming two posterior branches with subparallel sides, each with two appendages: a submedian short, straight, glabrous one and a thin folioform stalked appendage protruding backward. Folioform appendage about 0.6 of length of plate. Plate glabrous, distal appendages with somewhat granular surface.

##### Distribution.

China (Tibet), Indonesia (Sulawesi).

##### Remarks.

*Samarangopus canalis* was originally described and only known from Sulawesi Island, Indonesia (Scheller 2009). The anal plate, the male genital papillae as well as the protuberances on the body of Chinese specimens are nearly the same with *S. canalis* which proved the species identity. The main difference is that the posterior branches of anal plate of Chinese specimens each have two appendages, with a submedian, short, straight, glabrous appendage present, but absent in the types from Sulawesi. Other minor differences are the body size, numbers of protuberances on the body and the lengths of setae, bothriotricha, and flagella, which might belong to the variances between populations of different localities. In addition, the anal plate of *S. canalis* and *S. zhongi* sp. nov. both having two appendages, but the shape of posterior one is different: bladder is triangular in *S. zhongi* sp. nov. but folioform in *S. canalis*.

## Supplementary Material

XML Treatment for Samarangopus

XML Treatment for Samarangopus zhongi

XML Treatment for Samarangopus canalis
